# Phenotypic and functional characterization of synovial fluid-derived fibroblast-like synoviocytes in rheumatoid arthritis

**DOI:** 10.1038/s41598-021-01692-7

**Published:** 2021-11-12

**Authors:** Ditte Køster, Johanne Hovgaard Egedal, Søren Lomholt, Malene Hvid, Martin R. Jakobsen, Ulf Müller-Ladner, Hermann Eibel, Bent Deleuran, Tue Wenzel Kragstrup, Elena Neumann, Morten Aagaard Nielsen

**Affiliations:** 1 Department of Biomedicine, Aarhus University, Høegh-Guldbergs Gade 10, 8000 Aarhus, Denmark; 2 Department of Clinical Medicine, Aarhus University, Aarhus, Denmark; 3 Department of Internal Medicine and Rheumatology, Justus-Liebig-University Giessen, Campus Kerckhoff, Bad Nauheim, Germany; 4 Department of Rheumatology and Clinical Immunology and Center for Chronic Immunodeficiency, Medical Center and Faculty of Medicine, University of Freiburg, Freiburg, Germany; 5 Department of Rheumatology, Aarhus University Hospital, Aarhus, Denmark

**Keywords:** Immunology, Inflammation, Chronic inflammation

## Abstract

Fibroblast-like synoviocytes (FLS) play an important pathological role in persistent inflammatory joint diseases such as rheumatoid arthritis (RA). These cells have primarily been characterized in the RA synovial membrane. Here we aim to phenotypically and functionally characterize cultured synovial fluid-derived FLS (sfRA-FLS). Paired peripheral blood mononuclear cells (PBMC) and sfRA-FLS from patients with RA were obtained and monocultures of sfRA-FLS and autologous co-cultures of sfRA-FLS and PBMC were established. The in situ activated sfRA-FLS were CD34-, CD45-, Podoplanin+, Thymocyte differentiation antigen-1+. SfRA-FLS expressed uniform levels of NFкB-related pathway proteins and secreted several pro-inflammatory cytokines dominated by IL-6 and MCP-1. In a co-culture model with autologous PBMC, the ICAM-1 and HLA-DR expression on sfRA-FLS and secretion of IL-1β, IL-6, and MCP-1 increased. In vivo*,* human sfRA-FLS were cartilage invasive both at ipsilateral and contralateral implantation site. We conclude that, sfRA-FLS closely resemble the pathological sublining layer FLS subset in terms of surface protein expression, cytokine production and leukocyte cross-talk potential. Further, sfRA-FLS are comparable to tissue-derived FLS in their capabilities to invade cartilage at implantation sites but also spread tissue destruction to a distant site. Collectively, sfRA-FLS can serve as a an easy-to-obtain source of pathological sublining FLS in RA.

## Introduction

Rheumatoid arthritis (RA) affects approximately 1% of the adult population^1,2^. Despite being a systemic disease, the primary clinical manifestations are synovitis and joint destruction. RA fibroblast-like synoviocytes (RA-FLS) play a central role in initiation and persistence of both synovitis and joint damage^3,4^. In RA, the RA-FLS acquire a pathogenic phenotype that includes a persistently activated NFκB pathway, local proliferation, production of proinflammatory cytokines and chemokines, and cartilage invasion^3,5,6^. As a result of inflammation in the synovial membrane, effusion of cell-rich synovial fluid (SF) fills the joint cavity. Both the synovial membrane as well as the SF have been shown to contain RA-FLS^7^. The pathogenic phenotype is maintained in RA-FLS cultures up to passage 7, regardless of the cells being removed from the inflamed microenvironment, enabling ex vivo culturing of RA-FLS^8,9^.

Recent analyses of synovial tissue with single-cell resolution have identified multiple subsets of RA-FLS which can be divided into two main distinct disease-associated subpopulations; a lining layer and a sublining layer subtype of RA-FLS^10,11^. The lining- and sublining layer RA-FLS seem to have distinct roles in the modulation of cytokine production and joint destruction^10^. Especially, the podoplanin (PDPN)^+^ Thymocyte differentiation antigen-1 (THY1)^+^ CD34^−^ subset of RA-FLS located in the sublining layer undergoes a dramatic expansion and is associated with increased disease activity in RA^10^. These PDPN^+^THY1^+^CD34^−^ sublining layer RA-FLS display a unique pathogenic phenotype which is linked to HLA-DR expression^11^. Further, the expression of ICAM-1 on these cells has been shown to be selectively increased on RA-FLS compared with FLS from psoriatic arthritis^12^. These RA-FLS (PDPN^+^THY1^+^CD34^−^) mediate increased cytokine production, leukocyte infiltration and activation^10,13–16^*.* Further, PDPN^+^THY1^+^CD34^−^ sublining layer RA-FLS may activate the lining layer RA-FLS. This may lead to amplified joint destruction^10,17^. Owing to these pathophysiologic properties, RA-FLS subsets are of significant interest as novel therapeutic targets^17,18^. However, the accessibility of these pathological RA-FLS is limited and no alternative commercial cell line is currently available^19,20^.

Increased effusion of SF into the joint cavity is a hallmark of RA allowing RA-FLS to be cultured from the SF. Thus, the SF may be used as an alternative to tissue biopsies as a source of RA-FLS^20^. This potential also takes advantage of the the fact that SF aspiration is a part of routine RA treatment. However, the function and phenotype of SF-derived FLS (sfRA-FLS) have not yet been well characterized and compared with the recently well characterized tissue-derived FLS (tRA-FLS) subsets.

Thus, in the present study we aim to phenotypically and functionally characterize sfRA-FLS and compare these to the well-established tRA-FLS subsets harvested from the synovial tissue, hereby evaluating on their potential in future RA-FLS related studies.

## Results

### Fibroblast-like synoviocytes derived from synovial fluid are phenotypically homogeneous and different from non-pathological fibroblasts at passage 4

Fibroblast-like synoviocytes (FLS) were cultured from synovial fluid mononuclear cells (SFMC) from RA patients during a disease flare. In 80% of the included RA patients, a homogenious population of proliferating cells displaying fibroblast-like morphology were obtained at passage 4. The cells were passaged with trypsinization to eliminate both non-adherent cells and strongly adherent cells between each passage^21^ (Supplementary Fig. S1a–d).

To phenotypically characterize this population, the expression of surface markers (PDPN, THY1 and CD34), cytokine and chemokine production as well as expression of proteins of the NFκB signaling pathway were analyzed.

**Figure 1:**
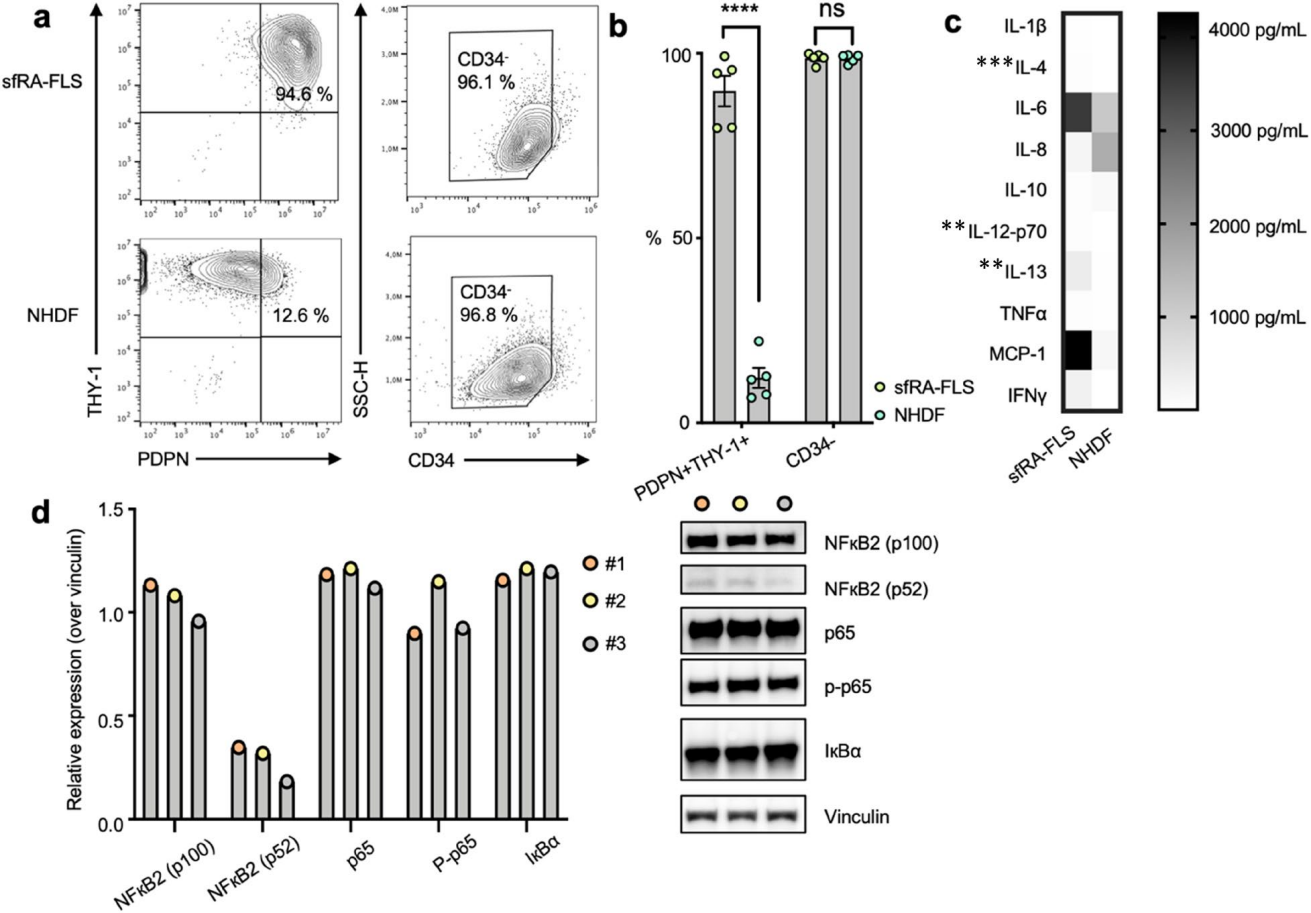
Characterization of surface markers, cytokine production and expression of NFκB pathway related proteins by synovial fluid-derived fibroblast-like synoviocytes (sfRA-FLS). (**a**) Expression of PDPN, THY-1 and CD34 by CD45^neg^ sfRA-FLS (top) and NHDF (bottom). Data shown from representative donors (**b**) Percentage of PDPN^+^THY-1^+^ and CD34^-^ CD45^neg^ sfRA-FLS (n = 5) and NHDF (n = 5). Data are represented as mean ± SEM. (**c**) Heatmap representing cytokine secretion by sfRA-FLS (n = 5) and NHDF (n = 3–5) cultured for 48 h. Data represented as mean. (**d**) Western blot analysis of NFκB related pathway proteins by 3 untreated sfRA-FLS donors (#1, #2, #3), full-length blots/gels are presented in Supplementary Fig. S5. Protein expression levels normalized to vinculin. sfRA-FLS, Synovial-fluid derived fibroblast-like synoviocytes; NHDF, Normal human dermal fibroblast; PDPN, Podoplanin; THY-1, Thymocyte differentiation antigen 1; SSC-H, side-scattered light (height); IL, interleukin; TNF, tumor necrosis factor; MCP-1, Monocyte chemoattractant protein-1; IFN, interferon; NF-κB, nuclear factor kappa-light-chain-enhancer of activated B cells; IκBα, nuclear factor of kappa light polypeptide gene enhancer in B-cells inhibitor.

The CD45^-^ sfRA-FLS were predominantly PDPN^+^THY1^+^ double positive (89.8% ± 9.2%) and CD34^−^ (98.7% ± 0.6%) (Fig. 1a and supplementary Fig. S2).

The sfRA-FLS phenotype were compared with normal human dermal fibroblasts (NHDF) displaying significantly less PDPN^+^THY1^+^ double positive cells, but comparable levels of CD34^−^ expression (Fig. 1b). Further, the uniform population of sfRA-FLS produced multiple proinflammatory cytokines without exogenous stimulation, markedly exceeding the secretion from NHDF (Fig. 1c and Table 1). The MCP-1 and IL-6 secretion from sfRA-FLS were 16 and 2.5 times higher than the secretion from NHDF, respectively (Fig. 1c). Furthermore, IL-4, IL-12 and IL-13 from sfRA-FLS was significantly higher than the secretion from NHDF, the same pattern applied to the majority of other inflammatory cytokines measured but due to donor variations and a limited sample size, significant differences were not identified in all (Table 1).

**Table 1:** Cytokine and MCP-1 production (pg/mL) by NHDF (n = 3–5), sfRA-FLS (n = 5) and CD3 and CD28 activated PBMC (n = 5) in mono-culture and by sfRA-FLS and activated PBMC in co-culture (n = 5).

	Mono-culture NHDF	Mono-culture sfRA-FLS	Mono-culture PBMC (activated)	Co-culture
IL-1β	2.57 (0.67–5.3)	4.5 (3.9–7.8)	30.2 (14.3–43-5)	383.1 (146.5–523.5)
IL-4	0.77 (0.47–1.15)	27.7 (21.7–44.2)	38.2 (26.5–55.6)	684.5 (80.6–5033)
IL-6	1033 (526.7–1759)	2648 (1327–6116)	172.7 (89.96–363.8)	18,150 (highest cut-off value)
IL-8	2264 (138.7–2814)	243.9 (101.1–315.6)	13,875 (11,480-highest cut-off value)	13,875 (highest cut-off value)
IL-10	0.49 (0.001–276.5)	39.1 (30.3–62.4)	106.2 (75.9–152.7)	130 (59.5–175.5)
IL-12-p70	1.91 (1.27–2.66)	21.5 (18.0–46.2)	31.7 (22.2–50.0)	507.9 (106.8–7062)
IL-13	12.79 (5.6–22.0)	432.3 (224.7–543.5)	633.2 (521.5–833.5)	1719 (621.1–4727)
TNFα	5.49 (0.96–29.44)	23.4 (8.8–51.0)	628.7 (337.9–944.7)	530.2 (284.2–941.5)
MCP-1	151 (105.5–205.9)	2458 (691.7–8719)	3.9 (3.9–1982)	65,103 (14,347–100,000)
IFNγ	2.31 (1.50–3.78)	7.8 (3.9–718.6)	5898 (3046–21,868)	7835 (4725–21,700)

Data represented as median with 25–75% percentile. IL, interleukin; TNF, tumor necrosis factor; MCP-1, monocyte chemoattractant protein 1; IFN, interferon; sfRA-FLS, Synovial-fluid derived fibroblast-like synoviocytes, PBMC, peripheral blood mononuclear cells; NHDF, normal human dermal fibroblasts. ND = not determined.

In support of the uniform population of passage 4 sfRA-FLS, we detected similar levels of NFκB-related pathway proteins across different RA donors (Fig. 1d). Together, these results indicate that sfRA-FLS cultures at passage 4 are not only morphologically, but also phenotypically homogeneous, and functionally active by releasing several inflammatory cytokines dominated by MCP-1 and IL-6 without exogenous stimulation.

### Synovial fluid derived FLS cross-talk with *autologous* PBMC and increase secretion of pro-inflammatory cytokines

We next examined the cross-talk potential of this morphological and phenotypical uniform sfRA-FLS population. Since ICAM-1 and HLA-DR are expressed by tRA-FLS and linked to their inflammatory properties, the expression of ICAM-1 and HLA-DR on sfRA-FLS were measured accordingly. The expression was evaluated both on unstimulated and stimulated cells. The cells were stimulated with either TNFα, IFNγ or co-cultured with anti-CD3/CD28-activated autologous PBMC.

**Figure 2:**
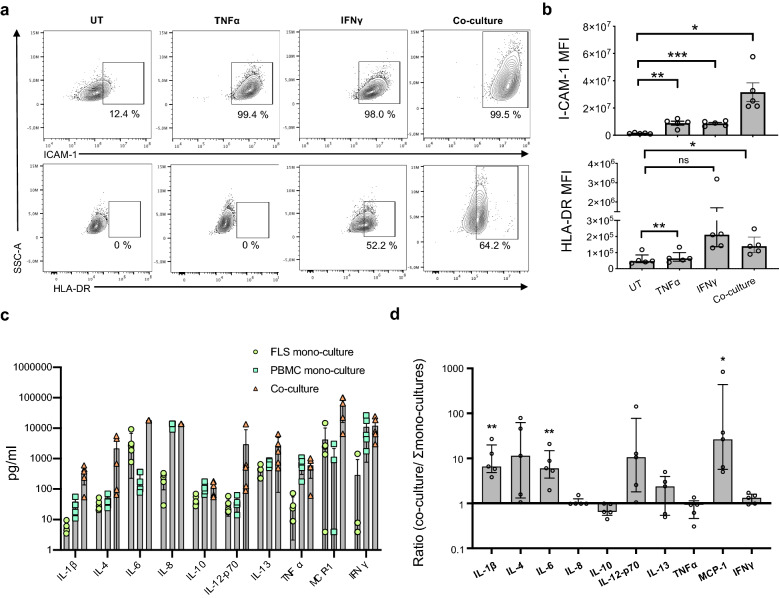
sfRA-FLS and PBMC cross-talk affects expression of surface markers and cytokine expression by sfRA-FLS. (**a**) Expression of ICAM-1 (top) and HLA-DR (bottom) by sfRA-FLS UT or after stimulation with 10 ng/mL TNFα or 10 ng/mL IFNγ or after co-cultivation with activated autologous PBMC for 48 h. Data shown from representative donors. All cells were pre-gated on the CD45^−^CD34^−^PDPN^+^THY-1^+^ population. (**b**) MFI values of ICAM-1 (top) and HLA-DR (bottom) expression by sfRA-FLS (n = 5) as in (**a**). ICAM-1 data is represented as mean with SEM and HLA-DR data is represented as median with interquartile range. (**c**) Cytokine secretion by sfRA-FLS (n = 5) and PBMC (n = 5) mono-cultures and sfRA-FLS + PBMC co-cultures (n = 5) cultured for 48 h. (**d**) As in (c) but cytokine secretion by co-cultures is normalized to the summarized cytokine secretion by sfRA-FLS and PBMC mono-cultures. Values above 1 represent an increased cytokine secretion in co-cultures compared to the total production in mono-cultures. Data represented as median with interquartile range.&&& *p ≤ 0.05, **p ≤ 0.01. UT, untreated; ICAM-1, intercellular adhesion molecule-1; HLA, human leukocyte antigen; TNF, tumor necrosis factor; IFN, interferon. IL, interleukin; MCP-1, monocyte chemoattractant protein 1; SSC-A, Side-scattered light (area); MFI, mean fluorescence intensity; sfRA-FLS, Synovial-fluid derived fibroblast-like synoviocytes, PBMC, peripheral blood mononuclear cells: ns, not significant.

SfRA-FLS increased the expression of ICAM-1 by 7.2 ± 1.5 (TNFα), 6.5 ± 0.7 (IFNγ) and 22.5 ± 2.9 (co-culturing) fold (*p* = 0.008, *p* = 0.0004 and *p* = 0.01). Likewise the expression of HLA-DR increased by 1.2 ± 0.04 (*p* = 0.003) (TNFα), 16.2 [2.2;36.3] (*p* = ns) (IFNγ), and 2.9 ± 0.7 (*p* = 0.04) (co-culturing) fold upon stimulation with TNFα or IFNγ or after co-cultivation (Fig. 2a,b).

Next, the effect of sfRA-FLS cross-talk with anti-CD3/CD28-activated autologous PBMC were evaluated by analyzing the differences in cytokine production between mono- and co-cultures. Higher amounts of IL-1β, IL-6 and MCP-1 were secreted from co-cultures compared with the summarized production from monocultures of sfRA-FLS and activated PBMC (*p* = 0.003, *p* = 0.008, and *p* = 0.02) (Fig. 2c,d, and Table 1). Interestingly, a tendency towards decreased IL-10 secretion in co-cultures compared with the total secretion from monocultures of sfRA-FLS and activated PBMC was observed.

### Immortalized RA human synoviocytes

Immortalized RA human synoviocytes (^IM^FLS) from 2 different RA donors^22^ were also examined, to determine their similarities with sfRA-FLS and hereby their potential in RA studies. ^IM^FLS were predominantly PDPN^+^THY1^+^ double positive (84.4%; 73.6–95.1%) and CD34^−^ (99.7% ; 99.4–99.9%) (Fig. S3). In addition, ^IM^FLS secreted high levels of IL-6 that were increased following either TNFα or INFγ stimulation (Fig. S4a).

The expression of ICAM-1 and HLA-DR on ^IM^FLS also markedly increased after stimulation with TNFα or IFNγ respectively (Fig. S4b, c) comparable to the expression seen on sfRA-FLS, although ^IM^FLS displayed a higher unstimulated expression of both ICAM-1 and HLA-DR compared with primary sfRA-FLS.

### SfRA-FLS are able to invade human cartilage implants both ipsilateral and contralateral

**Figure 3:**
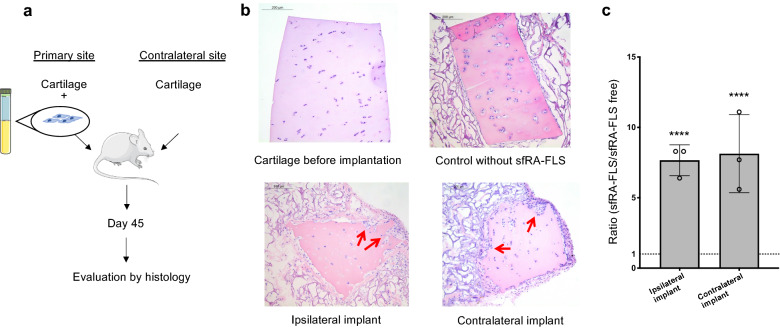
sfRA-FLS are cartilage invasive and have abilities to migrate to unaffected joints. Cartilage invasion of sfRA-FLS at primary implant and at contralateral implant in SCID mice after 45 days. (**a**) Cartilage and sfRA-FLS were co-implanted at primary site into SCID mice and cartilage without sfRA-FLS were implanted at contralateral site. Results were compared to SCID mice with only cartilage implants at ipsilateral and contralateral site (sfRA-FLS free). Cartilage invasion score were evaluated at day 45. 0 = no invasion, 3 = more than 10 cell depths invasion. sfRA-FLS were isolated and cultured from RA donors (n = 3) and repeated with 5 mice (n = (6 groups × 5 animals) 30). (**b**) Representative histology showing cartilage invasion (red arrows) in both ipsilateral implant and contralateral implant. (**c**) Ratio describing sfRA-FLS invasion in ipsilateral and contralateral implants normalized to sfRA-FLS free SCID mice. Data is represented as median with interquartile range, ****p ≤ 0.0001. Figures were generated with images from Servier Medical Art (www.servier.com), licensed under the Creative Commons Attribution 3.0 Unported License (http://creativecommons.org/license/by/3.0/).

Finally, we investigated the invasive capacity of human sfRA-FLS in vivo. Human cartilage and sfRA-FLS were co-implanted in a carrier matrix into SCID mice at the primary co-implantation site subcutaneously and cartilage without sfRA-FLS in a carrier matrix at the contralateral site (Fig. 3a). At day 45 co-implanted sfRA-FLS were found to be cartilage invasive at both the primary and contralateral implantation site (Fig. 3b). Invasive scores of ipsilateral implants were 1.7, (1.3–1.7) and contralateral implants were 1.5 (1.1–2.2). The invasion score of sfRA-FLS at both the primary and contralateral sites were more than 5.6-fold higher compared to control implants (animals receiving carrier matrix with cartilage but without sfRA-FLS) (*p* < 0.0001 and *p* < 0.0001) (Fig. 3c). These findings were comparable to earlier publications using tRA-FLS^23^. These results indicate that human sfRA-FLS are capable of invading cartilage in vivo and have the ability to systemically migrate to the contralateral site and be cartilage invasive also in a distant location.

## Discussion

**Figure 4:**
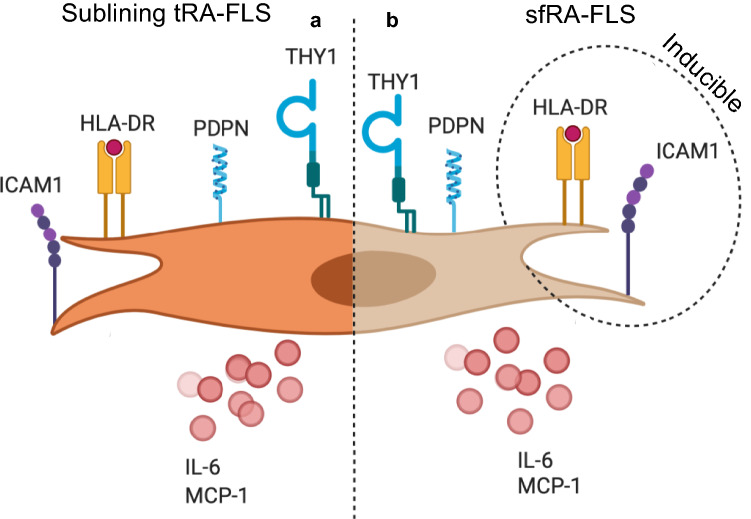
Similarities of pathological FLS harvested from the sublining layer or synovial fluid in RA. (**a**) A selection of subtype specific surface markers with corresponding dominated cytokine secretion. In general characterized by an upregulation of inflammatory pathways (transcriptomic profiling). (**b**) FLS harvested from the synovial fluid of flaring RA patients passaged in vitro displaying similar surface markers constantly or inducible, compatible cytokine secretion and increased joint destruction. Created with BioRender.com.

In recent years, subsets of human RA-FLS have been extensively characterized leading to the discovery of pathological FLS subsets. Particularly, the PDPN^+^THY1^+^CD34^−^ sublining layer FLS subset is robustly linked to RA pathology^10,17,18^ (Fig. 4). The SF has been proposed as an alternative source of RA FLS^20^. Following the recent advances in the understanding and subclassification of tRA-FLS, a characterization of sfRA-FLS is needed to facilitate the understanding of results generated by these cells and potentially supplement the use of tRA-FLS^18,19^.

Interestingly, after passaging, the sfRA-FLS cultures consisted of primarily PDPN^+^THY1^+^CD34^−^ FLS. This subset of pathological FLS is present in the synovial fluid at passage 0 but at this passage only constitutes a small fraction of all sfRA-FLS^24^. Hence, the aggressive and proliferative nature of these cells causes them to outgrow other sfRA-FLS subsets during in vitro cultivation. At passage 4, we observed a homogenous culture of around 90% pathological sfRA-FLS. These results show, that the majority of passage 4 sfRA-FLS are undistinguishable from the pathogenic subset of tRA-FLS, specifically with respect to receptor expression and inflammatory cytokine production^4,10,11^ (Fig. 4). Hence, the present study supports that synovial fluid, harvested as part of routine treatment, can be used to acquire and expand pathological FLS utilizing a trypsinization passaging protocol^7,20^. These PDPN^+^THY1^+^CD34^−^ sfRA-FLS secrete multiple proinflammatory cytokines and display a uniform basal expression of NFκB pathway-related proteins across different RA donors.

These results also underline the persisting pathogenic, inflammatory and aggressive phenotype of sfRA-FLS conserved through passaging in the majority of chronic RA patients with active disease even despite ongoing treatments. In addition to an increased production of proinflammatory cytokines, HLA-DR expression were increased on sfRA-FLS after co-culture with either immune cells or addition of exogenous INFγ. This demonstrates that sfRA-FLS have the capacity to engage in direct antigen presentation, supporting the findings by Tran et al.^14^.

Further, sfRA-FLS have the ability to invade cartilage at the site of inflammation but also to migrate to and invate human cartilage at a distant site, similar to tRA-FLS previously described by Lefévre et al.^23^. This potential could be confirmed recently with a mixed population of adherent SFMC, however, our data suggest that this capacity is mainly, if not solely, mediated by the pathological sfRA-FLS subset^7^. Whether this cartilage invasiveness is direct or through a secondary activation of the surrounding tissue remains to be clarified^18^. However, that the sfRA-FLS without positional identity could potentially inherit both proinflammatory and tissue-destructive properties, as reported recently by Kevin Wei et al.^17^. Therefore, the distinction between lining and sublining layer FLS in RA may not be fixed and could potentially change due to tissue localization, cytokine milieu or extracellular matrix proteins, a plasticity known from synovial macrophages neighbouring FLS in the lining and sublining layer^17,25,26^.

In the present study, the majority of samples isolated from active RA patients gave rise to proliferating sfRA-FLS cultures. The fact that a fraction of RA SFMC could not form these proliferating cultures may reflect differences in the cellular composition of the inflamed joint^7,27,28^. Notably, we detected no differences between patient laboratory parameters or treatment with respect to their capabilities to form proliferating sfRA-FLS cultures (Table 2). Despite previous results showing that the treatments had different efficacy in sfRA-FLS cultures^20,28^. Additionally, ^IM^FLS from two RA donors showed phenotypic and functional similarities with the sfRA-FLS. To our knowledge, this subset characterization of ^IM^FLS is the first showing similarities between two human RA cell lines, harvested from either synovial tissue or synovial fluid, and sublining pathological PDPN^+^THY1^+^CD34^−^ FLS.

**Table 2:** Patient characteristics.

Patient characteristics (n = 9)	n = 7	n = 2
Disease duration (years):	11.5 (4–21.3)	18.5 (1–36)
DAS28CRP (0–10) :	4.2 (3–5.3)*	2.6 (2.3–3)
**Serology (%)**		
Sero positive/Sero negative:	60/40**	100/0
**Gender (%)**		
Male/female	71/29	50/50
**Treatment at time of therapeutic arthrocentesis (%)**		
Mono TNFi	17***	0
Mono MTX	33	50
TNFi + MTX	50	50

Shown as donors who either were able to (n = 7) or failed to (n = 2) form sfRA-FLS cultures. Data are expressed as %. *Three patients had missing DAS28CRP data **Two patients had missing serology data ***One patient had missing treatment data. DAS28CRP, disease activity score 28 based on CRP; RA, rheumatoid arthritis.

The present study also describes how sfRA-FLS, resembling pathological FLS, can be obtained and purified from patients with active RA. A limitation to the use of these sfRA-FLS, is that SF is primarily collected from a selected group of RA patients with large joint involvement. Further, SF can usually only be obtained from flaring patients. Thus, sfRA-FLS isolation is mostly relevant for studies on chronic flaring RA patients with large joint involvement. However, the cells from these patients, namely the treatment non-responders, are of particular interest, since a large proportion of this group has previously been shown to have fibroblast-dominated local pathology^27–29^. Further, after passage 4, sfRA-FLS have proliferated to an extent that allows for the establishment and analysis of autologous co-culture models eliminating the potential confounding factors that may arise from allogenic culturing^30^.

It would be of interest to further compare cells acquired from synovial biopsies with synovial fluid samples in future studies to clarify the potential of sfRA-FLS as a minimal invasive way of stratifying patients to a more personalized treatment^28,30^. Moreover, whether sfRA-FLS share properties with the recently described non-adherent preinflammatory mesenchymal (PRIME) cells found in increased numbers systemically in RA patients preceding disease flares^30^ would also be of key interest to address in future projects.

The present phenotypical and functional characterization of sfRA-FLS, acquired and activated at the site of pathology, will facilitate an improved understanding of the results generated by the use of these cells in various ex vivo and in vivo FLS models. In combination with ^IM^FLS cell lines, these FLS models may increase our understanding of the role and transformation of pathological FLS in RA. The FLS purification approach and later cultivation may be applicable to a wide range of other inflammatory joint diseases including osteoarthritis, in which SF are accessible.

## Materials and methods

### Patients and samples

A cross-sectional, paired set of PBMC and SFMC were obtained from patients with chronic RA (n = 9), with at least one swollen joint, at the outpatient clinic at Aarhus University Hospital, at the time of therapeutic arthrocentesis. Out of these nine RA patients, 7 donors were able to provide proliferating and viable FLS cultures at passage 4. Blood and synovial fluid were collected in EDTA tubes. Patient characteristics are described in Table 2. Immortalized RA FLS (^IM^FLS) were kindly provided by Prof. Hermann Eibel^22^.

### Ethics

All human samples were obtained after informed written consent according to the Declaration of Helsinki and approved by the Local Ethics Committee (The Central Denmark Region committee on health research ethics, project number 20121329; ethics committee Giessen, Germany, 66/08 and 74/05) and the Danish Data Protection Agency. All animal experiments were carried out following the guidelines of the German Animal Welfare Act.

### Cell isolation and cultivation

PBMC and SFMC were isolated from blood and synovial fluid, respectively, by conventional Ficoll-Paque (GE HealthCare, Chicago, IL, USA) density gradient centrifugation and cryopreserved at -150 °C until time of analysis.

PBMC were cultivated in RPMI (Lonza, Walkersville, MA, USA), 10% fetal calf serum (FCS), 1% penicillin (Lonza), 1% streptomycin (Lonza), 1% HEPES (Gibco, Thermo Fisher Scientific, Waltham, MA, USA) and 1% glutaMAX (Gibco) (later termed RPMI full medium) in a humidified 37 °C CO_2_ incubator. SFMC, sfRA-FLS, ^IM^FLS and normal human dermal fibroblasts (NHDF) were cultivated in DMEM (Lonza) full medium.

NHDF originated from foreskin of healthy individual donors undergoing circumcision. The foreskin biopsy was obtained at the Department of Urology at Aarhus University Hospital, Skejby, Denmark and primary foreskin fibroblasts were isolated from the tissue. Immediately after collection, the biopsies were placed in a 100 mm Petri dish and rinsed in 75% ethanol. Any subcutaneous fat or capillaries were removed and the tissue was cut into pieces of 5 × 5 mm to dissociate epidermis and dermis. The tissue pieces were incubated overnight in 10% RPMI medium supplemented with 2.4 U/ml Dispase II (Sigma-Aldrich). To isolate fibroblasts, the biopsies were then incubated in 10% RPMI supplemented with 2 mg/ml collagenase type IV-S (Sigma-Aldrich) and 200 U/ml DNase I (Sigma-Aldrich) for 3–6 h with occasional mixing. The digestion was inactivated with FCS and the tissue was disrupted by vortexing for 30 s. Then the cell suspension was filtered through a 70–100 µm nylon cell strainer and centrifuged at 200xg for 5 min. Supernatant was discarded and the cells were washed before subsequent culture in DMEM with 10% FCS, 1% L-glutamine and 1% penicillin and 1% streptomycin and cryopreserved at -150 °C.

### Synovial fluid derived RA-FLS cultivation

SfRA-FLS were isolated by culturing SFMC for 48 h in DMEM full medium whereupon non-attached cells were washed away with PBS and remaining cells were cultured in a humidified 37 °C CO_2_ incubator in DMEM full medium. When cells were > 70% confluent they were washed in PBS and subsequently passaged using trypsin–EDTA treatment (Gibco), as previously described by Nielsen et al.^30^. SfRA-FLS were used for analysis at passage 4 for ex vivo experiments and up to passage 7 for in vivo experiments.

### Cytokine stimulation of sfRA-FLS and ^IM^FLS

SfRA-FLS and ^IM^FLS were seeded (n = 20,000) in a Nunc™ 48-well plate (Thermo Fisher Scientific) and cultured until > 70% confluent. Next the fibroblasts were stimulated with 10 ng/mL TNFα (Peprotech, Rocky Hill, NJ, USA) or 10 ng/mL IFNγ (Peprotech) in fresh media for 48 h before media were removed and safed at -20 °C and cells were harvested for flow cytometry analysis as previously described^31^.

### Autologous sfRA-FLS and PBMC co-cultures

Autologous RA PBMC (n = 0.5 × 10^6^) were seeded in a Nunc™ 48-well plate (Thermo Fisher Scientific) and activated with Dynabeads® Human T-Activator CD3/CD28 (Thermo Fisher Scientific) in a 1:2 bead to cell ratio for 48 h in RPMI full medium supplemented with 15 µg/mL gentamicin in a humidified 37 °C CO_2_ incubator. Beads and supernatants were removed after 48 h of activation according to the instruction provided by the manufacture and cells were resuspended in fresh DMEM full medium supplemented with 15 µg/mL gentamicin and transferred to wells with > 70% confluent autologous sfRA-FLS and cultured for additionally 48 h. PBMC monocultures were cultured in wells without sfRA-FLS for 48 h following 48 h activation.

### Light microscopy

Cells were examined by bright-field microscopy using Olympus IX71 and pictures were obtained using Leica DFC350F.

### Western blot

Cells from 3 different sfRA-FLS donors (n = 1.5 × 10^5^) were lysed in respectively 75 µl and 25 µl Pierce RIPA lysis buffer (Thermo Scientific), supplemented with 10 mM NaF, 1X complete protease cocktail inhibitor (Roche) and 0.25 IU mL^−1^ benzonase (Sigma). Whole-cell lysates were denatured for 5 min at 95 °C in the presence of Laemmli Sample Buffer, 2X (Sigma) at a dilution of 1:2 before loading on gel. Separation was done by 10% SDS-PAGE gel electrophoresis (Criterion TGX gels, Bio-Rad). Gel was run for 1.5 h at 90 V and subsequently transferred onto a PVDF membrane using a Trans-Blot-Turbo transfer system for 7 min. Membrane was blocked for 1 h at room temperature with 5% skim milk (Sigma-Aldrich) in TBS with 0.05% Tween-20 (TBST). The membrane was cut into smaller pieces and incubated overnight at 4 °C with any of the following specific primary antibodies in TBST with 5% BSA: anti-NFκB p65 (Cell Signaling #8242, 1:1000), anti-phospho-NFκB p65 (Cell Signaling #3303, 1:1000), anti-NFκB p100/p52 (Cell Signaling #3017, 1:1000), anti-IκBα (Cell Signaling #4814, 1:1000) and anti-Vinculin (Sigma-Aldrich #V9131, 1:7500) used as loading control. After three washes in TBST, secondary antibodies, peroxidase-conjugated F(ab)2 donkey anti-mouse IgG (H + L) (1:10,000) or peroxidase-conjugated F(ab)2 donkey anti-rabbit IgG (H + L) (1:10,000) (Jackson Immuno Research) were added in TBST with 5% skim milk for 1 h at room temperature. After three additional washes in TBST, the membranes were exposed on an Image Quant LAS4000 mini imager (GE Healthcare) using Clarity Western ECL Blotting Substrate (Bio-Rad) or the SuperSignal West Femto maximum sensitivity substrate (Thermo Scientific). Protein levels were quantified by densitometry using the online Image J software.

### Flow cytometry

Fibroblasts were blocked with 0.1 mg/mL mouse IgG (Jackson ImmunoReseach) and 0.1 mg/mL rat IgG (Jackson ImmunoReseach) for 15 min and stained with CD34-PerCP-eFlour710 (Clone: 4H11, Thermo Fisher Scientific), CD45-APC-Cy7 (Clone: HI3, Biolegend, San Diego, CA, USA), THY-1-PE-Cy7 (Clone: 5E10, Biolegend), Podoplanin-PE (Clone: NZ-1.3, eBioscience, Thermo Fisher Scientific), ICAM-1-BV421 (Clone: HA58, BD Bioscience, Franklin Lakes, NJ, USA), HLA-DR-BV650 (Clone: L243, Biolegend) and fixable LIVE/DEAD nIR Dead Cell Stain Kit (Life Technologies, Thermo Fisher Scientific). Data were acquired on NovoCyte Quanteon (ACEA Bioscience inc., San Diego, CA, USA) and processed in FlowJo (FlowJo software version 10.5.3).

### ELISA and mesoscale

Supernatants from monocultures of NHDF, sfRA-FLS, PBMC, ^IM^FLS and sfRA-FLS + PBMC co-cultures were collected and stored at − 20 °C until analysis. MCP-1 and IFNγ levels in supernatants were assessed by ELISA MAX™ Deluxe Set Human MCP-1/CCL2 or IFNγ (Biolegend) and levels of IL-1β, -2, -4, -6, -8, -10, -12-p70, -13 and TNFα were assessed by V-PLEX Proinflammatory Panel 1 Human Kit (Meso Scale Diagnostics, Rockwille, MD, USA). Experiments were performed according to the manufacturer’s protocol and samples were diluted 1:2.

### SCID mouse model of RA

Female immunodeficient Crl-scidBR mice (Charles River, Germany) were housed under standardized conditions under pathogen-free conditions with water and food ad libitum. Animals underwent inverse-wrap implantation^32^. In brief, subcutaneous implantation of human sfRA-FLS together with cartilage (intact cartilage areas from osteoarthritis patients) in a Gealfoam carrier matrix (Pfizer, USA) was performed at the primary ipsilateral side. Contralaterally, cartilage without sfRA-FLS was implanted. Implants were removed after 45 days, snap frozen and 5 µm sections H/E-stained for scoring^32^. Invasion score: 0 = no invasion, 1 = visible invasion (2 cell depths), 2 = invasion (5 cell depths), 3 = deep invasion (> 10 cell depths). The animal study and evaluation was performed in compliance with the ARRIVE guidelines. The outcome measure was the fibroblast-mediated cartilage invasion and scoring of invasion was performed by blinded scorers. All animals were allocated to the respective groups by randomisation and there were no animals excluded from the study.

### Statistics

Statistical analyses and graphs were done using GraphPad Prism 7 for Mac (GraphPad Software). Normally distributed data are represented as mean ± SEM and were analyzed by Student’s paired t-test. Non parametric data are represented as median [25%;75% percentile] and were analyzed by Wilcoxon signed-rank test. Data were transformed to ratios by dividing the value of the samples with the value of the matched control. A two-sided *p* value < 0.05 was considered statistically significant.

### Ethical approval and consent to participate

All samples were obtained after informed written consent according to the Declaration of Helsinki and approved by the Local Ethics Committee (The Central Denmark Region committee on health research ethics, project number 20121329; ethics committee Giessen, Germany, 66/08 and 74/05) and the Danish Data Protection Agency. Animal experiments were approved by the local committee of the regional council in Hesse, Germany (15-B2/275).

## Conclusion

SfRA-FLS closely resemble the pathological sublining layer FLS subset in terms of surface protein expression, cytokine production and cross-talk potential. Further, sfRA-FLS are comparable to tRA-FLS, specifically with respect to their capability to invade cartilage at implantation sites but also to spread tissue destruction to distant locations. Collectively, models using sfRA-FLS can serve as an alternative source of pathological FLS in RA.

## Supplementary Information

Supplementary Information.

## Data Availability

The datasets used and/or analysed during the current study are available from the corresponding author on reasonable request.

## Abbreviations

BSA: Bovine serum albumin
CD: Cluster of differentiation
DAS28CRP: Disease activity score 28 based on C-reactive protein
ELISA: Enzyme-linked immunosorbent assay
FCS: Fetal calf serum
FLS: Fibroblast-like synoviocytes
HLA-DR: Human leukocyte antigen- DR isotype
ICAM-1: Intercellular adhesion molecule 1
IFNγ: Interferon gamma
Ig: Immunoglobulin
IL: Interleukin
^IM^FLS: Immortalized RA human synoviocytes
MCP-1: Monocyte chemoattractant protein 1
MTX: Methotrexat
NF-κB: Nuclear factor kappa-light-chain-enhancer of activated B cells
NHDF: Normal human dermal fibroblasts
PB: Peripheral blood
PBMC: Peripheral blood mononuclear cells
PBS: Phosphate-buffered saline
PDPN: Podoplanin
PRIME: Preinflammatory mesenchymal
RA: Rheumatoid arthritis
RA-FLS: Rheumatoid arthritis fibroblast-like synoviocytes
RF: Rheumatoid factor
SF: Synovial fluid
SFMC: Synovial fluid mononuclear cells
SfRA-FLS: Synovial fluid-derived fibroblast-like synoviocytes
TBS: Tris-Hcl buffered saline
TBST: Tris-Hcl buffered saline with 0.05% Tween-20
THY1: Thymocyte differentiation antigen-1
TNFα: Tumor necrosis factor alpha
TNFi: Tumor necrosis factor alpha inhibitor
tRA-FLS: Tissue-derived fibroblast-like synoviocytes
UT: Untreated
