# Supplementary material for: Phenotypic and functional characterization of synovial fluid-derived fibroblast-like synoviocytes in rheumatoid arthritis
Source: Sci Rep. 2021 Nov 12;11:22168. doi: 10.1038/s41598-021-01692-7 (PMC8590001; doi:10.1038/s41598-021-01692-7)
Supplement: Supplementary file 1 — Supplementary Information. [file 41598_2021_1692_MOESM1_ESM.pdf]

Supplementary Figure S1.

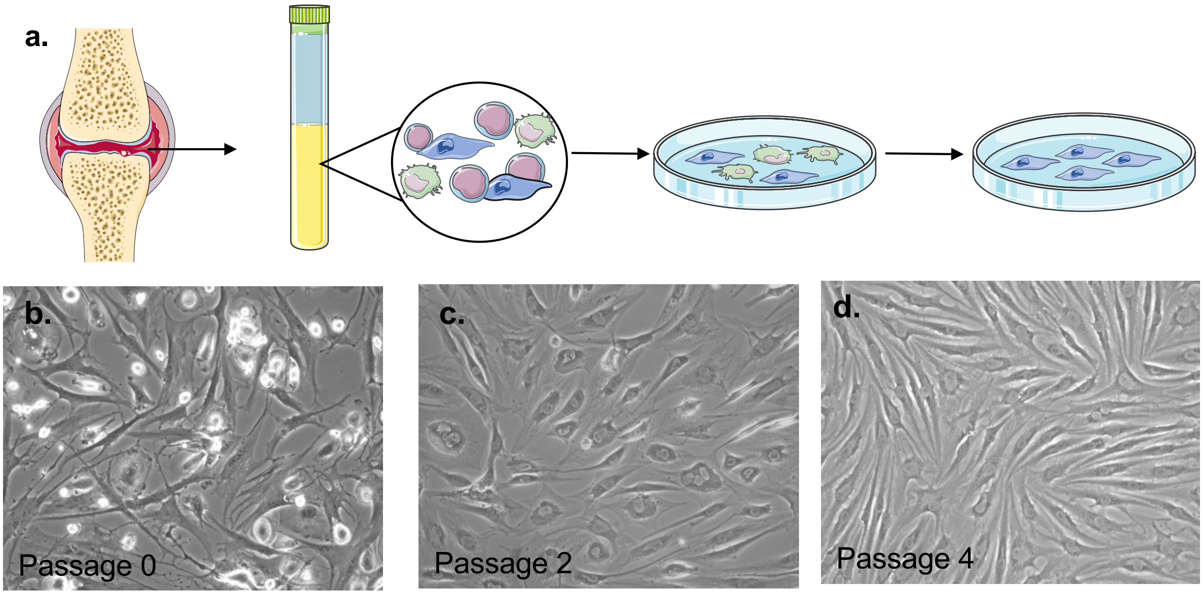

Figure S1. Fibroblast-like synoviocytes (FLS) can be cultured from mononucleated cells derived from synovial fluid of rheumatoid arthritis patients. Pure FLS cultures (<2 % contamination) were obtained by a strict trypsinization method between passages to exclude well-attached cells like monocytes. **(a)** Schematic representation of the workflow. Figures were generated with images from Servier Medical Art (www.servier.com), licensed under the Creative Commons Attribution 3.0 Unported License (http://creativecommons.org/license/by/3.0/).
(b) In primary cultures (passage 0) a mixed population of branched spindle-shaped cells, large round cells and polynucleated cells were observed after 2-3 weeks of culturing compatible with sfRA-FLS, macrophages and osteoclasts, respectively. **(c)** After passage 1, a more uniform population of branched spindle-shaped cells exhibiting fibroblast-like morphology were observed. **(d)** At passage 4, a homogenious population of cells displaying fibroblast-like morphology were observed.

Supplementary figure S2.

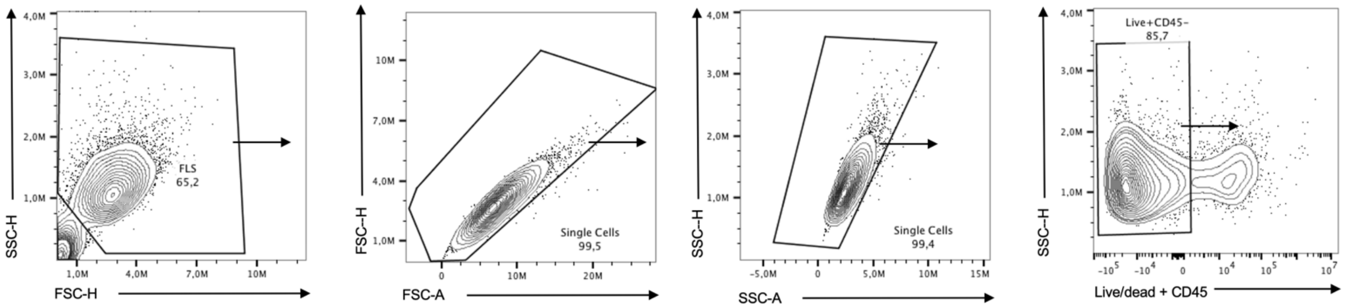

Figure S2. Gating strategy for all fibroblasts. A representative gating strategy prior to analysis of sfFLS, NHDF and ^{IM}FLS illustrating living CD45^{-} single cells.

Figure S3.

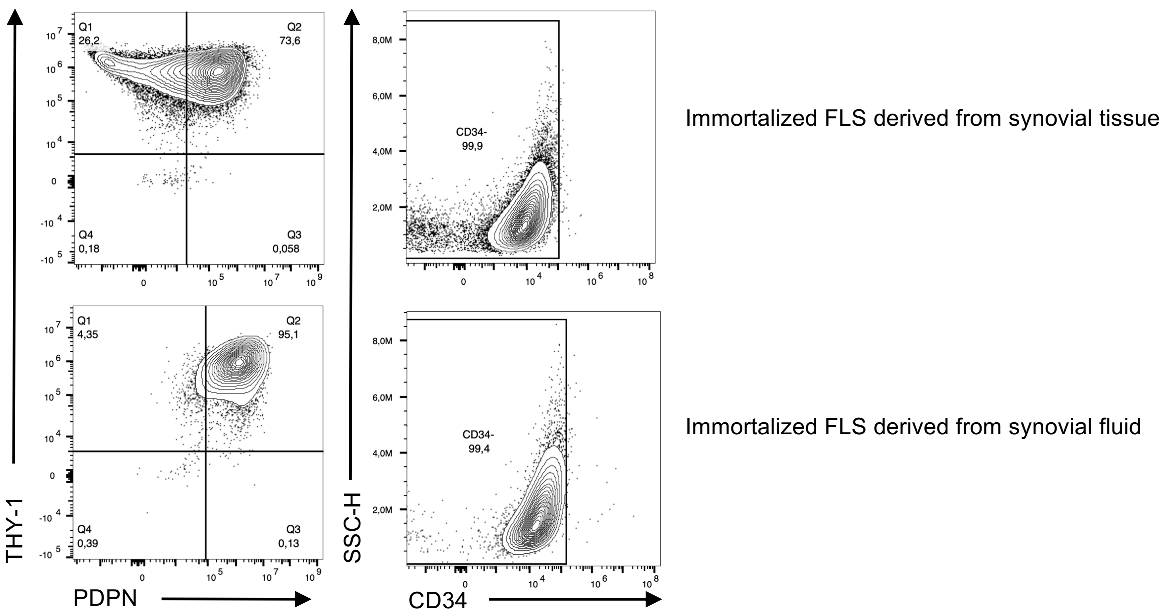

Figure S3. Expression of PDPN, THY-1 and CD34 by UT CD45^{neg} gated immortalized human synoviocyte cell lines derived from either synovial tissue (top) or synovial fluid (bottom). FLS, fibroblast-like synoviocyte; PDPN, Podoplanin; THY-1, Thymocyte differentiation antigen 1; SSC-H, side-scattered light (height).

Figure S4.

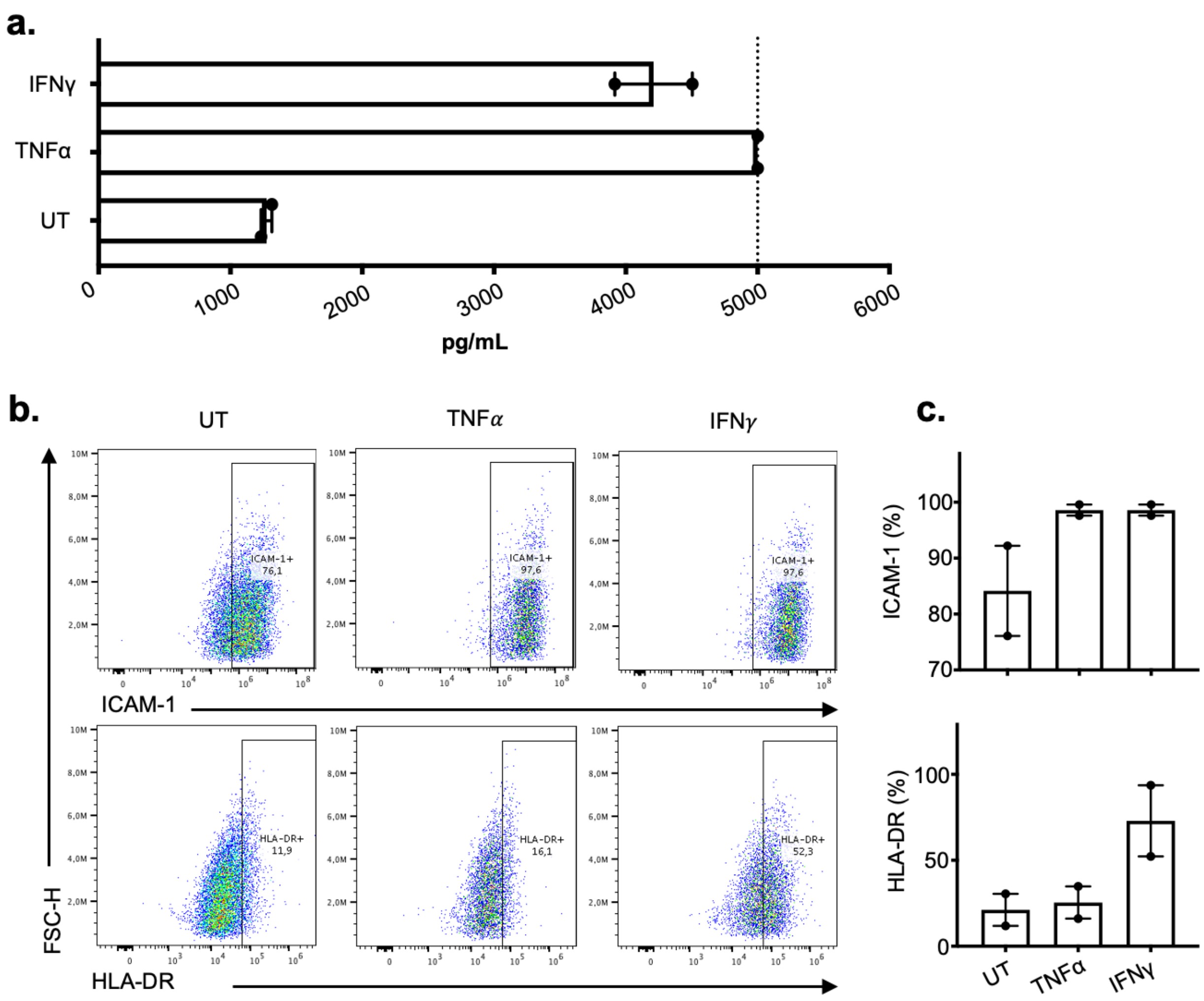

Figure S4. (a) IL-6 production by immortalized human RA synoviocyte cell lines either UT or treated with 10 ng/mL TNFα or 10 ng/mL IFNγ. Maximum detection level were 5000 pg/mL. **(b)** ICAM-1 (top) and HLA-DR (bottom) expression by CD45-CD34-PDPN+THY-1+ gated immortalized human RA synoviocyte cell lines either UT, treated with with 10 ng/mL TNFα or 10 ng/mL IFNγ. Data shown from one representative donor. **(c)** Percentage expression of ICAM-1 (top) and HLA-DR (bottom) as in (a) (n=2). Data represented as median with interquartile range. UT, untreated; ICAM-1, intercellular adhesion molecule-1; HLA, human leukocyte antigen; TNF, tumor necrosis factor; IFN, interferon; FSC-H, Forward-scattered light (height).

Figure S5.

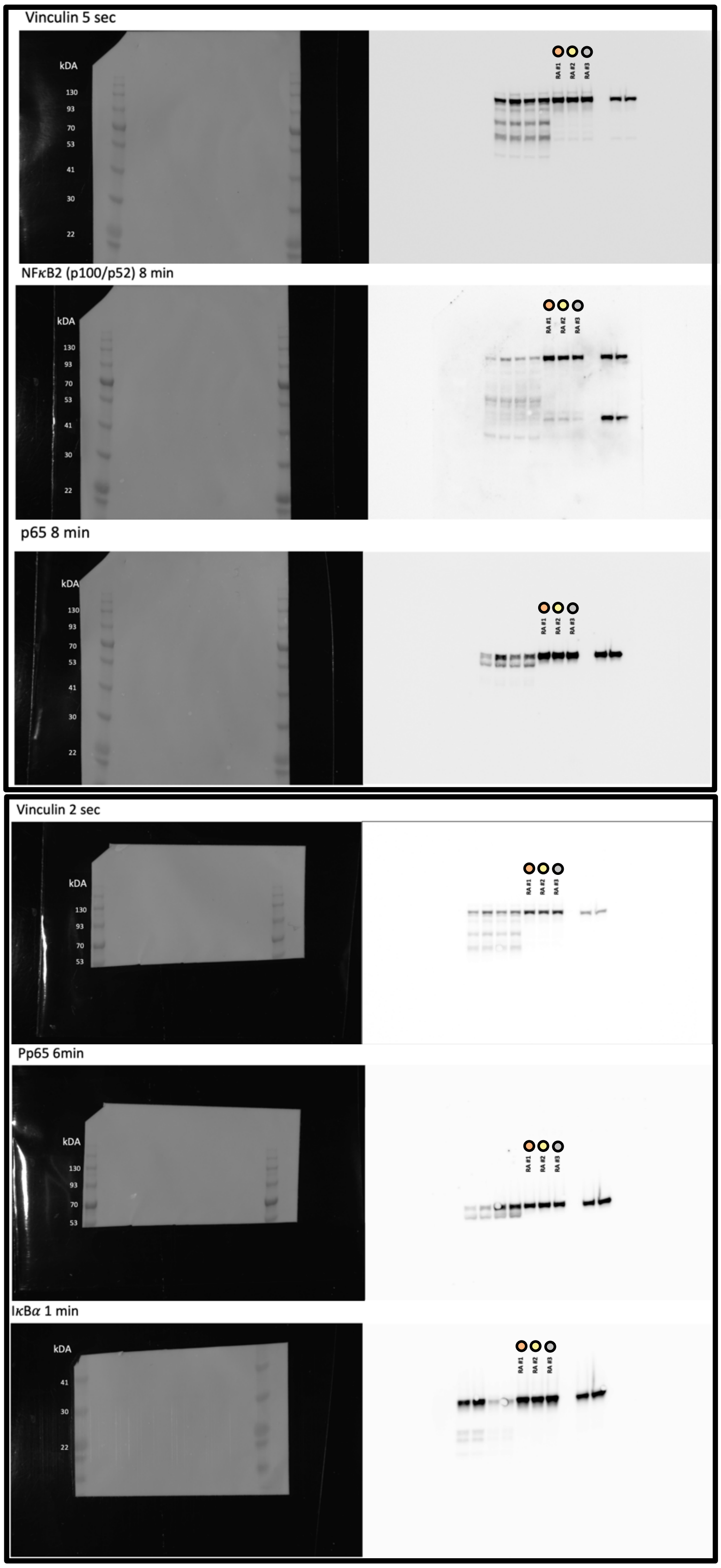

Figure S5. Full-length blots/gels of the Western blot analysis of NFκB related pathway proteins by 3 untreated sfRA-FLS donors (#1, #2, #3) presented in Figure 1d. NFκB Protein expression levels normalized to vinculin blot in the same bl
